# PROTOCOL: Efficacy and effectiveness of micronutrient supplementation and fortification interventions on the health and nutritional status of children under‐five in low and middle‐income countries: a systematic review

**DOI:** 10.1002/CL2.196

**Published:** 2018-10-18

**Authors:** Emily C Keats, Aamer Imdad, Jai K Das, Zulfiqar A Bhutta

## Background

### The problem, condition or issue

Micronutrients are essential vitamins and minerals that are obtained from the diet and are required for everyday cellular functioning [Bailey 2015]. Micronutrient malnutrition (MNM), or the absence of these key vitamins and minerals, continues to be a pervasive problem in low‐ and middle‐income countries (LMICs) today, especially in infants and children. In these settings, MNM can occur in several ways: i) from insufficient nutrient intakes due to low consumption of foods or a lack of dietary diversity, ii) from inhibited absorption of nutrients due to infections, inflammation, or chronic disease [Bailey 2015], or from both. As a result, diets are not able to meet the requirements for rapid growth, making children under‐five one of the groups most vulnerable to MNM [Bailey 2015]. Globally, it is estimated that 43% of children under‐five have anaemia [Stevens 2013]; approximately 42% of these cases are amenable to iron supplementation (i.e. caused by an iron deficiency) [Stevens 2013]. 29% of children aged 6‐59 months are deficient in vitamin A [Stevens 2015], 30% of school‐age children have insufficient iodine intakes [Andersson 2012], and 17% of the population is at risk of inadequate zinc intake [Wessells 2012], though each of these estimates reflects a relatively large degree of uncertainty. Assessing nutritional status, through anthropometrics, clinical signs and symptoms, biochemical or dietary methods, is resource‐intensive and requires standardized definitions and tools to be comparable across countries. This task is especially challenging for infants and children under‐five, which has created gaps in micronutrient status data among these population subsets for several countries. Where country‐level data is available, it may not be up to date or appropriately disaggregated by relevant factors (e.g. region, sex, wealth) that could have important implications for intervention potential.

Multiple deficiencies frequently occur simultaneously, especially in food insecure situations that are more common in LMICs [Bailey 2015]. Micronutrient deficiencies by themselves, or in concert with overt malnutrition, can be associated with negative immediate and long‐term outcomes including physical, developmental, and cognitive consequences, increased susceptibility to infections, higher mortality, and poor health and productivity later in life [WHO 2001; Lozoff 2007; Sanghvi 2007; Black 2008]. For example, there is strong evidence to suggest that iron deficiency anaemia in infancy and pre‐school aged children results in poor motor development and potentially irreversible cognitive damage, with reduced learning and educational attainment later in life [Lozoff 2007; Christian 2010]. Iodine deficiency in childhood has also been shown to result in developmental delay [Walker 2011]. Vitamin A deficiency not only exacerbates mortality from common morbidities such as diarrhoea and measles, but it is also a risk factor for blindness among children [Imdad 2017]. Lastly, zinc deficiency has been associated with impaired growth and immune dysfunction, leading to stunting, wasting, and increased severity of infections [Mayo‐Wilson 2014; Brown 2009]. Together, undernutrition, including deficiencies of essential vitamins and minerals, stunting, and wasting, is estimated to cause 45% of deaths in children under‐five, resulting in 3.1 million deaths per year [Black 2013]. This review will focus on micronutrient supplementation during childhood.

### The intervention

This review will encompass several interventions that aim to address, directly or indirectly, the problem of MNM in children under the age of five.

1. **Micronutrient supplementation**
  Micronutrient supplementation includes the provision of single‐ (iron, vitamin A, zinc, iodine, vitamin D, vitamin B12, folic acid) or multiple‐micronutrients in the form of syrups, drops, tablets or capsules. Multiple micronutrient (MMN) supplements will be defined based on a composition of, at minimum, any three or more micronutrients [Kawai 2011].
2. **Lipid‐nutrient supplementation**
  Micronutrient supplementation can also take the form of lipid‐nutrient supplements (LNS), which provide energy, essential fatty acids, and protein along with micronutrients in a quantity of 20‐50 gm/day [Dewey 2012]. Small‐quantity LNS (SQ‐LNS), such as Nutributter, were designed for use in the home at a reduced quantity (20 gm/day) such that they would enrich elements of a typical diet as opposed to replacing all other foods [Dewey 2012].
3. **Large‐scale fortification**
  Large‐scale fortification refers to the process of adding one or more micronutrients to a staple food or condiment during central processing in order to improve nutritional quality. Staple items are chosen based on their widespread and regular consumption by the target population, and typically include flour, salt, sugar, oil, milk, and condiments.
4. **Targeted fortification for infants and young children**
  Targeted fortification is a practice that capitalizes on specific food vehicles for a subset of the population, in this case infant formula for infants less than 6 months of age and complementary foods or blended foods (e.g. infant cereal that contains rice, legumes, sugar and oil) for children over 6 months of age. Commercially available fortified complementary foods are distinct from complementary foods that have been fortified at the level of the household (see below).
5. **Point of use fortification with micronutrient powders**
  Multiple‐micronutrient powders are used to fortify foods at the point of use, typically in the home. They come in single‐use sachets that contain a combination of essential vitamins and minerals that can be sprinkled directly onto soft or semi‐solid foods [HF‐TAG 2016].

In general, supplementation interventions are used as preventive, short‐term strategies for specific population groups that are at risk of deficiency or they are used to treat MNM [Bailey 2015]. Supplements and single‐use sachets of micronutrient powders have the advantage of supplying the recommended nutrient intake per dose. Recommended schedules may be daily or intermittent (e.g. weekly or yearly), depending on the micronutrient.

Fortification strategies are more long‐term and have the capacity to reach a much broader audience if they are implemented successfully [Bailey 2015]. They are intended to be delivered at a population‐level, meaning the amount of micronutrient added to a food is governed by safety concerns relating to individuals at the higher end of consumption who could potentially exceed the tolerable upper intake level [WHO & FAO 2006]. As such, younger children who consume small quantities of food compared to adults may not reach the daily recommended intake of micronutrients from fortified foods alone.

### How the intervention might work

Because we aim to include both efficacy and effectiveness studies in this review and they answer two separate questions of biological potential and programmatic potential, respectively, we have split this section into a description of how the intervention might work for each.

Efficacy studies assess the biological potential of supplementation or fortification interventions under ideal conditions; the aim of each being to increase the micronutrient intake of the target population and improve downstream health outcomes. To do so, the micronutrient of interest must be ingested, absorbed, and bioconverted to the active compound within the human body in order to produce the desired health effects. The mechanism of action of each micronutrient is unique. For example, the absorption of iron by intestinal cells is regulated, responding to the body's need for iron: more iron is absorbed when there is increased demand, whereas there is diminished absorption when the need for iron decreases [Wheby 1963]. As such, absorption from supplements will depend on the baseline iron status of an individual. When iron is absorbed by intestinal cells, it can then be transported to the bone marrow and other tissues where it binds to receptors and is stored or used for biological functions. These functions range from tissue oxygenation and performance to neurotransmission to complex cognitive, psychological, and other physical functions of the body [Burhans 2005; Umbreit 2005]. In contrast, zinc is found in all cells of the human body and is required for every day cellular functioning through its enzymatic, structural (e.g. protein synthesis and folding), and regulatory (e.g. gene expression) roles [Tuerk 2009; MacDonald 2000; Hotz 2004; Stefanidou 2006; Aggarwal 2007; Hambidge 2007]. Zinc is also involved in the specific and non‐specific immune response [Shankar 1998], and in cellular growth, replication, and differentiation [MacDonald 2000; Stefanidou 2006]. This important role of zinc in immune function relates to the biological basis on which zinc supplementation may improve incidence of morbidities such as diarrhoea, lower respiratory tract infections, and malaria among children. Similar to zinc, vitamin A has immune properties that help to fight common infections such as measles and diarrhoea, particularly among children [Green 1928; Semba 1999; Imdad 2017]. In addition, vitamin A plays a role in essential biological processes that include growth, vision, red blood cell production, and reproduction [Sommer 1996]. Vitamin D is a fat‐soluble molecule that is transported to the liver via carrier proteins where it is converted to its circulating form, 25‐hydroxyvitamin D (25(OH)D) [Yakoob 2016]. 25(OH)D is further converted to its active form, 1,25‐dihydroxyvitamin D (1,25(OH)D), through enzymatic processes in the kidneys [Yakoob 2016]. The 1,25(OH)D will then bind to the vitamin D receptor, which is found on the nuclear membrane of a wide range of cell types, to regulate gene expression and other molecular mechanisms, particularly those relating to infection control and immune function overall [Cannell 2008; Gunville 2013]. For example, research on tuberculosis has indicated that exposure to 1,25(OH)D can induce the generation of antimicrobial peptides that can directly damage the cell membrane of *Mycobacterium tuberculosis* along with stimulating migration of monocytes [White 2008; Gunville 2013]. Iodine, though required in only minute amounts, is essential for the biosynthesis of thyroxine (T4) and triiodothyronine (T3), two thyroid hormones that are responsible for the regulation of metabolism, growth and development. Thus iodine deficiency has important implications throughout infancy and childhood, when mental and physical development occur simultaneously. The provision of multiple micronutrients, whether through supplementation regimens or micronutrient powders, has the advantage of providing several essential vitamins and minerals at once. Typically iron, folic acid, vitamin A, vitamin C, vitamin D, and zinc will be present and, depending on the manufacturer, the intervention could contain up to 15 micronutrients [De Pee 2009]. However, with a varied composition there is the potential for interactions between certain nutrients that could lead to poor absorption of other nutrients. It should be noted that data are available for a limited number of nutrients, limiting our ability to understand the potential for impact of several of the nutrients that are often included in fortified foods/supplements.

In fact, the potential to respond to micronutrient supplementation and fortification interventions will vary at an individual level. Bioavailability, broadly defined as the absorption and utilization of a nutrient, is influenced by a number of factors including those related to the host (age, sex, co‐morbidities or infections) as well as the nutrient's chemical form, its matrix for delivery, and other foods in the diet that could enhance or inhibit absorption [Krebs 2001]. There are several factors that apply specifically to paediatric populations, including increased nutrient requirements for growth and development, gastrointestinal tract maturation, the character of the diet, nutritional status, and growth rates during critical periods, that will impact bioavailability [Krebs 2001] and intervention efficacy. For children above 6 months of age (who have stopped exclusive breastfeeding), micronutrient interventions aim to supplement the diet in order to reach the recommended dietary allowance for a given nutrient. Primary studies and systematic reviews of randomised controlled trials (RCTs) in LMICs have provided proof‐of‐principle for each of these interventions, through improvement in both biochemical (e.g. serum micronutrient concentration) and functional (e.g. anaemia reduction, improved growth, reduced morbidity and mortality) health outcomes, in several cases [Andersson 2012; Imdad 2017; De‐Regil 2011; Mayo‐Wilson 2014; De‐Regil 2017; Gera 2017; Das 2013]. However, there is also the potential for adverse side effects of micronutrient interventions that could limit their acceptability, especially among children. For example, vitamin A supplementation can increase the risk of vomiting and bulging fontanelle among infants [Imdad 2017]. Furthermore, zinc and iron supplements have been associated with negative gastrointestinal side effects, such as vomiting.

**Figure 1:**
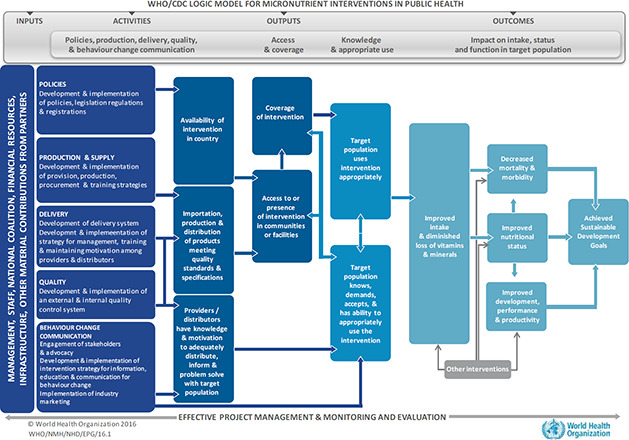
WHO/CDC logic model for micronutrient interventions in public health

Despite the biological plausibility of these interventions to improve micronutrient status and long term health outcomes, their success as public health interventions will rely on other factors. Logic models can help to better understand some of the push and pull factors that govern intervention effectiveness in the real world. Figure 1 represents the joint World Health Organization (WHO) and US Centre for Disease Control and Prevention (CDC) logic model for implementing micronutrient interventions in public health programmes [WHO & CDC 2011]. Logic models help to identify the pathway of effect between an intervention and an outcome, and also help to define measurable indicators throughout the process. While the model necessitates adaptation for local contexts, its theoretical underpinnings and processes can be applied to each intervention listed above. The model is structured around four major components: inputs, activities, outputs, and outcomes. Inputs are the resources required prior to the implementation of any intervention, such as financial resources, staff, materials and equipment, and basic infrastructure [De‐Regil 2014]. Activities comprise the actions that are necessary for initiation and to sustain an intervention. Activities include: a) the development of policies, legislation, and advocacy pertaining to the intervention, b) production and supply plans, c) establishing sustainable delivery systems, d) quality control mechanisms, and e) strategies for behaviour change and communication [De‐Regil 2014]. If implementation is successful, outputs will include improved coverage of or access to the intervention [De‐Regil 2014]. Lastly, outcomes represent the benefits achieved by that intervention, including knowledge and appropriate use by the target population, followed by impact on micronutrient intake, nutritional status, and long‐term functional health outcomes [De‐Regil 2014].

While this logic model is generic, it can be adapted to fit any micronutrient intervention, including those that aim to improve MNM in children under‐five. For example, in a recent paper, indicators for programme monitoring and surveillance of MMN supplementation in pregnancy were developed based on this WHO/CDC logic model, and found to be useful for designing and implementing surveillance systems in various settings [Mei 2017]. Because this review will go beyond collating data from controlled settings to include programmatic evidence, it will be important to consider this framework when interpreting the success or failure of an intervention to achieve impact. For instance, country context influences the development of policies relating to MNM and will also affect in‐country nutrition programming: two activities that have direct bearing on the availability of a supplementation or fortification intervention for children under‐five. Many low‐income countries, countries in conflict, or those recovering from environmental conditions such as drought or famine may be lacking in governmental and financial support for MNM because of resources that are directed elsewhere. In several of these situations, nutrition programmes, often operated through non‐governmental organizations or United Nations agencies, will largely dictate the supply and delivery of a particular intervention, thus influencing coverage and access to or presence of an intervention in communities. Additionally, factors that are more proximal to the child, including parental knowledge of an intervention and its appropriate use, are equally important for successful intervention uptake. Acceptance and compliance by caregivers, as well as children, is required for improved intake and status. Taken together, a review of programme implementation, including strength of implementation (whether fully implemented, partially, or not at all), programme quality/ functioning, and push and pull factors, will be critical to understand the effectiveness of an intervention and translate evidence for policy makers.

### Why it is important to do the review

This review will be instrumental in summarizing the available evidence on micronutrient interventions for children under‐five in low and middle‐income settings. This is a topic area that does not lack primary research; subsequently, many systematic reviews have been conducted on individual interventions (Appendix 1). However, several gaps remain that make this review a worthwhile endeavour.

Firstly, several of these reviews need updates to include the most recent trial findings. There is the hope that with additional data and a more robust sample, we will be better informed to interpret intervention effects that have, up to now, been inconclusive. For example, efforts to review the effects of vitamin D supplementation in children have been hampered by a limited number of trials that represented populations with varying degrees of vitamin D deficiency and were underpowered to evaluate mortality and other outcomes [Yakoob 2016]. Several recent vitamin D trials have been undertaken that would contribute to this evidence base [Gupta 2016; Somnath 2017]. Investigating the ‘ongoing studies’ section of other published reviews has revealed additional newly completed studies (e.g. a trial looking at the effect of point of use fortification of complementary foods on iron status, anthropometrics, and gut microbiota in Kenya [Tang 2017]), indicating a need to bring review data up to date wherever possible. Additionally, some outcomes have not been studied up until now. For example, the recent Cochrane review of vitamin A supplementation [Imdad 2017] examined morbidity and mortality outcomes, but did not look at growth among children. Lastly, there has been recent discussion about the role of zinc supplementation in improving child growth. Previous reviews have demonstrated conflicting results and significant heterogeneity in the pooled data [Brown 2009; Ramakrishnan 2009; Imdad 2011; Mayo‐Wilson 2014; Gogia 2012]. This review will attempt to understand these differences by performing subgroup analysis and meta‐regression to uncover reasons for the variability in effect of zinc supplementation for growth.

In addition to providing updated evidence from RCTs, this review will collate relevant data from evaluations of existing programmes for child under‐nutrition. Because of the multitude of exogenous factors that can influence a public health programme (e.g. interrupted production or supply, poor quality control, low intervention availability in certain geographies, or insufficient advocacy resulting in lack of behaviour change), plausible intervention benefits are not always translated into real life situations. With this added critical component of programme evaluation data to our review, we will be in a better position to provide recommendations of what interventions work in uncontrolled settings, and where gaps remain.

While there have been several efforts to collate data on individual interventions, the strength of this review lies in its comprehensive assessment of interventions to address MNM in children under the age of five years. We will examine a range of essential interventions using both trial and programmatic data. As stated above, efficacy and effectiveness studies ask two separate questions, both of which are important for policy and practice. Understanding an intervention's biological potential to improve health outcomes alongside the intervention's programmatic potential will allow policy makers to make more informed decisions based on a complete evaluation of an intervention's health impacts and relevance in a real world setting. As such, this review will be a ‘one‐stop shop’ for practitioners and policy makers who would like to see all the evidence for one type of intervention summarized in one place.

## Objectives

This review aims to summarize and synthesize the available evidence on micronutrient supplementation and fortification interventions for children under‐five in LMICs, including potential adverse effects of the interventions. A number of different interventions will be investigated, though each will be summarized separately.

### Specific objectives

1. What is the efficacy and effectiveness of micronutrient interventions (single or multiple micronutrient supplementation) on child health and nutritional status?
2. What is the efficacy and effectiveness of lipid‐based nutrient supplementation on child health and nutritional status?
3. What is the efficacy and effectiveness of targeted or large‐scale food fortification interventions on child health and nutritional status?
4. What is the efficacy and effectiveness of point of use fortification with micronutrient powders on child health and nutritional status?

## Methodology

### Criteria for including and excluding studies

#### Types of study designs

We will include primary studies, including large‐scale programme evaluations, which assess the efficacy and/or effectiveness of interventions using experimental and quasi‐experimental study designs that allow for causal inference. Efficacy studies, including RCTs and non‐randomised controlled trials, and effectiveness studies, including observational studies and programme evaluations, will be analysed separately.

► Studies where participants were randomly assigned, individually or in clusters, to intervention and comparison groups. Stepped‐wedge and cross‐over designs will be eligible for inclusion.
► Studies where non‐random assignment to intervention and comparison groups is based on other known allocation rules, including a threshold on a continuous variable (regression discontinuity designs) or exogenous geographical variation in the treatment allocation (natural experiments).
► Controlled before‐after studies in which allocation to intervention and control groups was not made by study investigators, and outcomes were measured in both intervention and control groups at baseline, and appropriate methods were used to control for selection bias and confounding, such as statistical matching (i.e. propensity score matching, or covariate matching) or regression adjustment (i.e. difference‐in‐differences, instrumental variables).
► Interrupted time series studies in which outcomes were measured in the intervention group at least three time points before the intervention was implemented and at least three time points after.

#### Types of participants

We will include studies conducted on healthy male and female children from 1 month up to 5 years of age living in any LMIC. Studies with children outside of this age range are eligible when we can disaggregate data for children aged 1 month to 5 years or where the overall (mean, median) age of participants lies between 1 month and 5 years of age. Because of differences in dietary requirements for children < 6 months of age and those ≥6 months of age, these age bands will be analysed and reported on separately.

Studies conducted on a specific group of pre‐identified “un‐healthy” children will be excluded. This includes studies that recruited diseased children (e.g. HIV‐infected), children with acute or chronic illnesses, as well as those that were anaemic, stunted, wasted, or malaria‐infected. We will include studies with apparently healthy children, however, the likelihood of children having micronutrient deficiencies at baseline is high due to the increased prevalence of MNM in these settings.

LMICs will be defined by the World Bank Group at the time when the study was conducted.

#### Types of interventions

The following interventions will be included, and will be analysed separately:

► Single micronutrient supplementation (iodine, iron, vitamin A, zinc, vitamin D, folic acid, and vitamin B12)
  ▷ Supplementation may take the form of tablets, capsules, drops, or syrup
► Iron‐folic acid supplementation
► MMN supplementation
  ▷ Studies that use fewer than three micronutrients in the composition of the MMN tablet will be excluded [Kawai 2011; Haider 2017]
► Lipid‐based nutrient supplementation
  ▷ Studies that assess the effects of LNS for the management of moderate or severe acute malnutrition will be excluded
► Large‐scale food fortification interventions (i.e. fortified staple foods and condiments)
  ▷ For inclusion, staple foods or condiments must be fortified with one or more of the following nutrients: iodine, iron, vitamin A, zinc, folic acid, vitamin D, or vitamin B12
  ▷ Staple foods will be defined in a context‐specific manner, based on population consumption habits (e.g. soy sauce is considered a staple across Asia
► Targeted fortification for infants 1‐5 months of age (i.e. formula)
  ▷ Formula‐based interventions are meant to replace breast milk and therefore provide all dietary requirements for infants; as such, these will be analysed separately from other targeted fortification interventions for older children that are intended to enhance nutrient intakes from regular diets
► Targeted fortification for children ≥6 month of age (i.e. complementary foods)
► Point of use fortification with micronutrient powders

We will include studies that address the prevention of micronutrient deficiencies and not the treatment; this is applicable to each intervention type.

There will be no restrictions regarding: i) duration of exposure to the intervention, ii) the provider of the intervention, iii) route of administration, iv) the frequency of the intervention (e.g. daily or intermittent supplementation), or v) the food vehicle utilized for LNS interventions. We will include studies where co‐interventions (e.g. de‐worming or education) are provided for both the intervention and the comparison groups.

Author‐defined comparison groups will be used for all analyses. Comparators may include inactive control interventions such as placebo, no treatment, or standard of care (e.g. usual feeding practices) or active controls where the comparison is the same type of intervention with a different composition of micronutrients (e.g. Iron‐folic acid supplementation compared to iron without folic acid or MMN with or without iron). The exception to this is the comparison of MMN supplementation to LNS, which is common and will be eligible. However, all meta‐analyses will be reported separately by comparison group (i.e. placebo and no treatment control data will not be pooled).

#### Types of outcome measures

To be included within this review, studies must have measured at least one of the following primary and/or secondary outcomes. Outcomes were chosen based on their relevance to both policy‐makers and practitioners, including health workers (doctors, nurses, community health workers, etc.), parents, and other caregivers.

***Primary outcomes***

1. All‐cause mortality
2. Cause‐specific mortality
  ► Diarrhoea
  ► Meningitis
  ► Measles
  ► Lower respiratory tract infection, including pneumonia
  ► Malaria
  ► Other
3. Nutritional status
  ► Anaemia prevalence [WHO 2011]
    ▷ Non‐anaemia: ≥110 g/L
    ▷ Anaemia: < 110 g/L
  ► Stunting (‐2 z‐score or lower)
  ► Wasting (‐2 z‐score or lower)
  ► Underweight (‐2 z‐score or lower)

***Secondary outcomes***

1. Morbidity (as defined by study authors), including all‐cause hospitalization
  ► Lower respiratory tract infection, based on one of the following definitions [Mayo‐Wilson 2014]:
    ▷ Difficulty breathing or rapid breathing, or both
    ▷ Difficulty breathing or cough, along with one or more of the following: age‐specific rapid breathing rates, lower chest wall in‐drawing, chest auscultation signs of pneumonia (decreased breath sounds, bronchial breath sounds, crackles, abnormal voice resonance, pleural rub), nasal flaring, grunting, fever, central cyanosis, inability to breastfeed or drink, vomiting everything, convulsions, lethargy, unconsciousness, or severe respiratory distress
    ▷ Clinical evidence of lower respiratory tract infection based on chest auscultation (decreased breath sounds, bronchial breath sounds, crackles, abnormal voice resonance, pleural rub) or chest radiograph
  ► Diarrhoea, defined as:
    ▷ Three or more loose stools per day
    ▷ One loose stool with blood
2. Micronutrient deficiencies
  ► Vitamin A (serum/plasma retinol)
  ► Iron (serum/plasma ferritin, plasma TfR)
  ► Haemoglobin concentration
  ► Serum/plasma folate
  ► Serum/plasma zinc
  ► Serum/plasma vitamin D
3. Growth
  ► Height (cm or height‐for‐age z‐score)
  ► Weight (kg or weight‐for‐age z‐score)
  ► Weight for height
  ► Head circumference
  ► Mid‐upper arm circumference
  ► BMI
4. Mental and motor skill development (as assessed by study authors e.g. Bayley Mental Development Index, Bayley Psychomotor Development Index, Stanford‐Binet Test, DENVER II Developmental Screening Test)
5. Adverse effects
  ► Gastrointestinal (vomiting, diarrhoea, stomach ache, constipation)
  ► Irritability
  ► Fever
  ► Headache
  ► Stained teeth
  ► Bulging fontanelle
  ► Kidney stones
  ► Other

#### Duration of follow‐up

There will be no restrictions regarding duration of follow‐up.

#### Types of settings

Other than LMIC criteria, there will be no restrictions regarding study setting.

### Search strategy

The search strategy will be guided by our PICO model (Table 1), but will not be restricted by outcome in order to retain a broader search. The search will be conducted using indexing terms, including medical subject headings (MeSH), keywords, and free text words. Details of the search strategy can be found in Appendix 2. The date restriction on published articles will be from 1995 to June 2018 in order to capture the most relevant evidence. Non‐English papers will be included only where trained personnel who speak that language can allow for in depth translation of information, including assessment of risk of bias. Languages for potential inclusion include: French, Spanish, and Persian (Farsi). Manual searches will be conducted within references lists of review articles and included studies, and experts will be contacted to obtain any additional relevant material that may have been missed. The search process, including month/year of search, will be documented to ensure that replication is possible.

**Table 1:** PICO elements used for formulating search strategy

**Elements**	**Concepts**
**Population**	Children aged 1 month to 5 years living in a low or middle‐income country ► LMIC defined at the time the study was conducted
**Intervention**	Micronutrient and energy supplementation interventions ► Single (iron, folic acid, iodine, vitamin A, vitamin D, vitamin B12, zinc) and multiple micronutrient supplementation (≥ 3 micronutrients) ► Lipid‐nutrient supplementation ► Large‐scale fortification of staple foods ► Targeted fortification for infants/young children ► Point‐of‐use fortification with micronutrient powders
**Control**	Author‐defined inactive control groups (i.e. placebo, standard of care, or no treatment) or active controls where the comparison is the same as the intervention with a different composition of micronutrients
**Outcomes**	Primary: ► All‐cause mortality ► Cause‐specific mortality (diarrhoea, meningitis, measles, lower respiratory tract infection, including pneumonia, malaria, or other) ► Nutritional status (anaemia, stunting, wasting, underweight) Secondary: ► Morbidity (lower respiratory tract infection, diarrhoea) ► Micronutrient deficiencies ► Growth (height, weight) ► Mental and motor development ► Adverse effects

### Electronic searches

***Database searches:***

The search will be run in the following databases, selected based on their applicability to the subject material:

► African Index Medicus
► CAB Abstracts
► CINAHL
► Cochrane Central Register of Controlled Trials (CENTRAL)
► Embase
► International Initiative for Impact Evaluations (3ie)
► LILACS (Latin American and Caribbean health sciences literature)
► MEDLINE
► WHO (IRIS, eLENA, regional databases)

***Unpublished studies:***

► clinicaltrials.gov
► ProQuest Dissertations & Theses Global
► WHO International Clinical Trials Registry Platform (ICTRP)

### Searching other resources

Non‐indexed, grey literature searches will be conducted to locate relevant programme evaluations and any additional trials. We will search Google and Google Scholar, along with websites of key international nutrition agencies such as the CDC, the Global Alliance for Improved Nutrition, HarvestPlus, Hellen Keller International, Nutrition International, Sight and Life Foundation, UNICEF, and the World Food Programme. Additionally, we will conduct grey literature searches within the Eldis, IZiNCG (for zinc studies), and Research for Development Outputs (R4D) databases.

### Description of methods used in primary research

We anticipate that the vast majority of included studies will be randomised or cluster‐randomised controlled trials that follow our inclusion/exclusion criteria, as listed above. For example, in a study published by Lundeen and colleagues [Lundeen 2010], children aged 6 to 36 months of age in Kyrgyz Republic were cluster‐randomised to an intervention arm or control arm. Intervention clusters received a daily sachet of micronutrient powder for 60 days, while the control clusters received no micronutrient powder until after study completion. Mean haemoglobin concentration was assessed at baseline and end‐line, to determine anaemia prevalence among both groups of children.

Because we are including programme evaluations and large‐scale fortification programmes (e.g. salt iodization) are now mandatory in many LMICs, we would also anticipate there to be several interrupted time series studies, whereby measurements of an outcome (e.g. urinary iodine concentration) are taken at the population level before and after the fortification implementation date. However, to meet our inclusion criteria, interrupted time series studies must have had three measurements taken before and after the event.

### Criteria for determination of independent findings

In order to take into account potential sources of dependency, we will group studies in terms of their location, population, the programme that is being evaluated (if applicable), and intervention type to ensure that there is no double counting of evidence when synthesizing results across studies. If there are multiple papers that describe the same trial, these will be combined and coded as a single study.

For trials that include multiple intervention arms, we will select one pair (intervention and control) that satisfy the inclusion criteria of the review and exclude the rest. If > 2 intervention groups meet the eligibility criteria, then these groups will be combined into a single pair‐wise comparison group and data will be disaggregated into corresponding subgroups, or these arms will be separated into different forest plots to ensure that there is no double counting of participants. Multiple outcome estimates within the same study will be analysed separately.

### Details of study coding categories

For all included studies, data will be abstracted into a standardized data abstraction form that is comprised of a general study information sheet and a quantitative outcomes sheet. All data abstraction will be performed in duplicate and any discrepancies would be resolved by discussion first, and then by involving a third reviewer.

Each general study information sheet will contain the following:

► General study information: authors, publication year, language of study, study design
► Study setting: World Bank region, country, World Bank income level, city/town, urban/urban slum/rural/mixed setting, duration of data collection, date of data collection
► Study population: sample size recruited, sample size analysed, male/female/mixed (%), age range of participants, mean/median age of participants, description of participants (i.e. inclusion/exclusion criteria applied to recruitment)
► Intervention characteristics: type of intervention, food vehicle utilized (where applicable), duration of intervention, level of delivery, unit of randomisation (where applicable), dose of micronutrient(s) provided, frequency of provision (i.e. daily, weekly, etc.), duration of follow‐up, attrition rate,
► Programmatic indicators (based on the WHO/CDC logic model): policies, production, delivery strategies, quality control, behaviour change communication strategies, access and coverage, knowledge and appropriate use
► Funding source of programme (where applicable)
► Quality assessment (see section below: critical appraisal of studies)

Each quantitative outcome sheet will contain the following:

► Subgroup (if applicable)
► Subgroup sample size
► Outcome type (based on outcomes listed above)
► Outcome units
► Outcomes:
  ▷ Outcome measure treatment group
  ▷ Outcome measure comparison group
  ▷ Standard deviation
► Effect size:
  ▷ Effect measure (specify type)
  ▷ 95% confidence interval
  ▷ P value of effect measure
  ▷ Standard error or t‐statistic

### Synthesis procedures and conventions

#### Selection of studies

Title/abstract screening will take place independently by two reviewers, using specified inclusion/exclusion criteria. Abstracts will be screened where not enough information can be gleaned from the title alone regarding whether to include (or exclude) the study for full text screening. All full texts will then be screened in duplicate, with application of the same inclusion/exclusion criteria. Any disagreements will be resolved by a third reviewer. Both title/abstract and full text screening will be done using Covidence, a web‐based software platform for systematic reviews. Inter‐reviewer reliability/agreement can be assessed by checking the number of conflicts in the Resolve Conflicts page following each stage of screening.

Examples of included studies:

► Osendarp, SJ et al. (2002) Effect of zinc supplementation between 1 and 6 mo of life on growth and morbidity of Bangladeshi infants in urban slums. Am J Clin Nutr; 76:1401‐8.
► Rivera, JA et al. (2010) Effectiveness of a large‐scale iron‐fortified milk distribution program on anemia and iron deficiency in low‐income young children in Mexio. Am J Clin Nutr; 91(2):431‐9.

Examples of excluded studies:

► Choudhary, N et al. (2012) Relative efficacy of micronutrient powders versus iron‐folic acid tablets in controlling anemia in women in the second trimester of pregnancy. Food Nutr Bull; 33(2):142‐9.
  ▷ Ineligible population (women of reproductive age)
► Hollar, D et al. (2010) Effect of a two‐year obesity prevention intervention on percentile changes in body mass index and academic performance in low‐income elementary school children. Am J Public Health; 100(4):646‐53.
  ▷ Ineligible population (high income country)
  ▷ Ineligible intervention (obesity prevention)

### Assessment of risk of bias in included studies

We will critically appraise individual studies using the Cochrane Effective Practice and Organisation of Care (EPOC) guidelines for randomised trials, non‐randomised trials, controlled before‐after studies, and interrupted time series studies. EPOC guidelines include the following standardized criteria for assessing bias of randomised, non‐randomised, and controlled before‐after studies [EPOC 2017]:

► Random sequence generation
► Allocation concealment
► Baseline outcome measurements similar
► Baseline characteristics similar
► Incomplete outcome data
► Knowledge of the allocated interventions adequately prevented during study
► Protection against contamination
► Selective outcome reporting
► Other risks of bias (e.g. bias in measurement: validity and reliability of the measures used)

For interrupted time series studies, the following criteria will be considered [EPOC 2017]:

► Intervention independent of other changes
► Shape of intervention effect pre‐specified
► Intervention unlikely to affect data collection
► Knowledge of the allocated interventions adequately prevented during study
► Incomplete outcome data
► Selective outcome reporting
► Other risks of bias (e.g. bias in measurement: validity and reliability of the measures used)

For programme evaluations, a careful assessment of implementation and quality will be undertaken.

All risk of bias assessments will be performed in duplicate. Any disagreements will be resolved by a third reviewer.

### Measures of treatment effect

For each outcome, data must be converted to the same format (e.g. means and standard deviations for continuous data), including appropriate conversion of scales such that an increase/decrease always indicates improvement or deterioration of an indicator.

Dichotomous and continuous outcomes will be analysed separately. For dichotomous outcomes, results will be presented as summary risk ratios with 95% confidence intervals in order to compare risk of the outcome between intervention and control groups. Risk ratios (events per child) and rate ratios (events per child year) will be combined for incidence data, because of their similar interpretation and scale. Continuous outcome data will be presented as either a mean difference, if outcomes have been measured on the same scale, or a standardized mean difference, if outcomes have been measured on different scales, with 95% confidence intervals. Both change from baseline scores and final measurements will be eligible, and can be pooled where there is meta‐analysis with mean difference (i.e. scales are the same and measurements are in the same unit) [Higgins 2011]. Careful consideration will be given to reporting of the appropriate means and standard deviations (either of final measurements or of changes from baseline) if both change and final values are used in one meta‐analysis. Final values and change scores will not be combined as standardized mean differences because the standard deviation in this case reflects differences in measurement reliability. Where it is necessary to combine measures of treatment effect with standardized mean differences, we will use change scores, given that the standard deviation of the change is also reported.

### Unit of analysis issues

Special attention will be given to cluster‐randomised trials; this is to ensure that clustering has been appropriately accounted for within the analysis of the primary study, such that study precision is not over or under‐estimated within our analysis. Effect estimates of cluster‐randomised trials can be adjusted using the mean cluster size (M) and the intra‐cluster correlation coefficient (ICC), which quantifies the extent to which data from the same cluster are correlated [design effect = 1 + (M‐1)*ICC]. The design effect is then used to adjust the study data. In this way, a trial will be reduced to its effective sample size. Using the same methods, we will also adjust for clustering within observational studies that have incorporated this design. We will not make any adjustments if authors have appropriately adjusted for clustering already.

Randomized and non‐randomised studies with contemporaneous comparison groups will be analysed separately, but may be pooled if differences in findings are not statistically significant. Findings from controlled before‐after and interrupted time series designs will be analysed and reported separately.

### Dealing with missing data

Where data are incomplete or in a form that cannot be converted with the information available, we will contact the corresponding author for clarification or to obtain missing data. If authors have accounted for missing data (i.e. multiple imputations), we will use the adjusted data within our analysis.

### Assessment of heterogeneity

Clinical and methodological heterogeneity will be explored by assessing the similarities and differences in included studies' participants, interventions, outcomes, and methods. Statistical heterogeneity will be assessed by visually inspecting forest plots, calculating the I^2^ statistic and conducting a Chi^2^ test, where a p value < 0.05 is considered statistically significant. In addition, we will report Tau^2^ for all random effects meta‐analyses conducted. Sources of heterogeneity will be explored using subgroup analysis (listed below).

### Assessment of reporting biases

If the number of studies is sufficient (> 10), funnel plots will be used to visually assess publication bias. This kind of bias is unlikely if data forms a symmetric inverted funnel shape around the mean effect estimate. In addition, we will perform Egger's test to assess funnel plot asymmetry.

### Data synthesis

Statistical analysis will be carried out using Review Manager 5.3 and Stata. For randomised controlled trials (RCTs), we will follow intention to treat analysis. If authors report a per protocol analysis, we will reconstruct the data to create an intention to treat analysis.

Random effects meta‐analysis will be used to account for any expected heterogeneity in interventions, comparisons, outcomes, or settings within the studies included in a given synthesis. Where meta‐analysis is deemed inappropriate due to substantial methodological or statistical heterogeneity between studies, the findings of the included studies will be summarized in narrative or table form.

The generic inverse‐variance approach will be used, such that adjustments to the study weights will be made according to the variance of the effect estimate (i.e. the larger studies with smaller standard error will be given more weight than smaller studies with larger standard error). For random effects analyses, the DerSimonian and Laird method will be applied to incorporate a measure of variation (Tau^2^) among intervention effects from different studies. Where possible, the most adjusted estimates will be used to construct meta‐analyses from all studies.

For interpretation of results, overall effect estimates that have an associated p value < 0.05 will be deemed statistically significant. Non‐significant findings will also be reported. Where possible, interaction tests will be used to determine if there is a relevant difference in effect across subgroups. Concluding that an intervention is effective in one subgroup but not another will be based on a direct test of the mean difference between the two groups (i.e. with meta‐regression).

**Quality of evidence**

We will use the GRADE tool to assess the body of evidence for each primary and secondary outcome reported in our review. The assessment will be summarized in a ‘Summary of Findings’ table, created with GRADE pro software.

GRADE employs five criteria to determine the quality of evidence [Atkins 2004]:

► Study limitations (e.g. in design and implementation that suggest bias)
► Consistency of effect
► Imprecision
► Indirectness
► Publication bias

There are three criteria that can upgrade the evidence [Atkins 2004]:

► Large magnitude of effect
► Presence of a dose response relationships
► Effect of plausible residual confounding

Additionally, there are five criteria that can downgrade evidence [Atkins 2004]:

► Risk of bias in individual studies
► Indirectness of evidence
► Unexplained heterogeneity or inconsistency of results
► Imprecision of results
► High probably of publication bias

Quality ratings, as determined by GRADE, are found in Table 2.

**Table 2:** Quality of evidence, as determined by GRADE criteria

**Quality**	Description
**Very low**	The true effect is probably markedly different from the estimated effect
**Low**	The true effect might be markedly different from the estimated effect
**Moderate**	The authors believe that the true effect is probably close to the estimate effect
**High**	The authors have a lot of confidence that the true effect is similar to the estimated effect

### Subgroup analysis and investigation of heterogeneity

Depending on data availability (≥3 studies), exploratory subgroup analyses will be conducted on the primary outcomes for the following variables, selected based on evidence to support their potential to impact the intervention effect:

► Age (1‐5 months, 6‐11 months, 12‐23 months, 24‐59 months)
► Gender
► Geographical region (based on WHO regions)
► Baseline nutritional status (anaemic vs. non‐anaemic, stunted vs. non‐stunted, underweight vs. normal weight)
► Duration of intervention or programme (short‐term (<3 months), medium‐term (3‐6 months), and long‐term (6‐12 months))
► Frequency of intervention (daily versus intermittent)

Results from subgroup analyses will be carefully interpreted. We will also use random‐effects meta‐regression techniques to assess how characteristics of studies (explanatory variables) may influence the size of the effect estimate (outcome variable). Potential variables may include the setting, dosing frequency, dosing form, compound, duration, gender, SES status, or nutritional status.

Any subgroup analysis that is conducted post‐hoc will be stated as such.

### Sensitivity analysis

Sensitivity analyses will be conducted to determine whether the removal of studies with high risk of bias or the removal of non‐randomised studies (for our assessment of efficacy) significantly impacts findings. We will define studies as having a high risk of bias if one or more domains have been judged as ‘high risk’ or two or more domains have been judged as ‘unclear risk’.

### Treatment of qualitative research

We do not plan to include qualitative research.

## Review authors

**Lead review author:**

**Table:** 

Name:	Emily C. Keats
Title:	Research Associate
Affiliation:	Centre for Global Child Health
Address:	686 Bay Street suite 11.9805
City, State, Province or County:	Toronto, ON
Post code:	M5G 0A4
Country:	CANADA
Phone:	416 813 7654 x 309518
Email:	emily.keats@sickkids.ca
**Co‐authors:**	
Name:	Aamer Imdad
Title:	Assistant Professor of Pediatrics
Affiliation:	State University of New York Upstate Medical University
Address:	Suite 504, 725 Irving Ave
City, State, Province or County:	Syracuse, NY
Post code:	13210
Country:	USA
Phone:	+1.315.447.5407
Email:	Aamer08@gmail.com
	
Name:	Jai Das
Title:	Assistant Professor
Affiliation:	Department of Paediatrics and Child Health, Aga Khan University
Address:	Stadium Road, PO Box 3500
City, State, Province or County:	Karachi, Sindh
Post code:	74800
Country:	Pakistan
Phone:	+92.21.3493.0051
Email:	Jai.das@aku.edu
	
Name:	Zulfiqar A. Bhutta
Title:	Co‐Director
Affiliation:	Centre for Global Child Health
Address:	686 Bay Street suite 11.9805
City, State, Province or County:	Toronto, ON
Post code:	M5G 0A4
Country:	CANADA
Phone:	416 813 7654 x 301774
Email:	Zulfiqar.bhutta@sickkids.ca

## Roles and responsibilities

Emily Keats, Aamer Imdad, and Jai Das have methodological, statistical, and information retrieval expertise. Zulfiqar Bhutta has content expertise. All additional team members will receive training in systematic review methods.

## Sources of support

Funding for this review came from a grant from the Bill & Melinda Gates Foundation to the Centre for Global Child Health at The Hospital for Sick Children (Grant No. OPP1137750).

## Declarations of interest

The authors are not aware of any conflicts of interest arising from financial or researcher interests.

## Preliminary timeframe

Approximate date for submission of the systematic review: February 2019

## Plans for updating the review

The corresponding author, Dr. Zulfiqar A. Bhutta, will be responsible for any forthcoming updates to the review.

## AUTHOR DECLARATION

### Authors' responsibilities

By completing this form, you accept responsibility for preparing, maintaining and updating the review in accordance with Campbell Collaboration policy. Campbell will provide as much support as possible to assist with the preparation of the review.

A draft review must be submitted to the relevant Coordinating Group within two years of protocol publication. If drafts are not submitted before the agreed deadlines, or if we are unable to contact you for an extended period, the relevant Coordinating Group has the right to de‐register the title or transfer the title to alternative authors. The Coordinating Group also has the right to de‐register or transfer the title if it does not meet the standards of the Coordinating Group and/or Campbell.

You accept responsibility for maintaining the review in light of new evidence, comments and criticisms, and other developments, and updating the review at least once every five years, or, if requested, transferring responsibility for maintaining the review to others as agreed with the Coordinating Group.

### Publication in the Campbell Library

The support of the Coordinating Group in preparing your review is conditional upon your agreement to publish the protocol, finished review, and subsequent updates in the Campbell Library. Campbell places no restrictions on publication of the findings of a Campbell systematic review in a more abbreviated form as a journal article either before or after the publication of the monograph version in Campbell Systematic Reviews. Some journals, however, have restrictions that preclude publication of findings that have been, or will be, reported elsewhere and authors considering publication in such a journal should be aware of possible conflict with publication of the monograph version in Campbell Systematic Reviews. Publication in a journal after publication or in press status in Campbell Systematic Reviews should acknowledge the Campbell version and include a citation to it. Note that systematic reviews published in Campbell Systematic Reviews and co‐registered with Cochrane may have additional requirements or restrictions for co‐publication. Review authors accept responsibility for meeting any co‐publication requirements.

**I understand the commitment required to undertake a Campbell review, and agree to publish in the Campbell Library. Signed on behalf of the authors**:

**Table:** 

**Emily Keats**	**16 October 2018**

## 1 Existing Reviews

**Micronutrient interventions:**

Imdad A, Mayo‐Wilson E, Herzer K, Bhutta ZA. Vitamin A supplementation for preventing morbidity and mortality in children from six months to five years of age. Cochrane Database Syst Rev 2017, Issue 3. Art. No: CD008524. doi:10.1002/14651858.CD008524.pub3.

De‐Regil LM, Jefferds ME, Sylvetsky AC, Dowswell T. Intermittent iron supplementation for improving nutrition and development in children under 12 years of age. Cochrane Database Syst Rev 2011, Issue 12. Art. No: CD009085. doi:10.1002/14651858.CD009085.pub2.

Sachdev H, Gera T, Nestel P. Effect of iron supplementation on mental and motor development in children: systematic review of randomised controlled trials. Public Health Nutr 2005;8(2):117‐32.

Mayo‐Wilson E, Junior JA, Imdad A, Dean S, Chan XH, Chan ES, Jaswal A, Bhutta ZA. Zinc supplementation for preventing mortality, morbidity, and growth failure in children aged 6 months to 12 years of age. Cochrane Database Syst Rev 2014, Issue 5. Art. No: CD009384. doi:10.1002/14651858.CD009384.pub2.

Brown KH, Peerson JM, Baker SK, Hess SY. Preventive zinc supplementation among infants, preschoolers, and older prepubertal children. Food Nutr Bull 2009;30(1):S12‐40.

Gogia S, Sachdev HS. Zinc supplementation for mental and motor development in children. Cochrane Database Syst Rev 2012 Dec 12;12:CD007991. doi:10.1002/14651858.CD007991.pub2.

Yakoob MY, Salam RA, Khan FR, Bhutta ZA. Vitamin D supplementation for preventing infections in children under five years of age. Cochrane Database Syst Rev 2016, Issue 11. Art. No: CD008824. doi:10.1002/14651858.CD008824.pub2.

Allen LH, Peerson JM, Olney DK. Provision of multiple rather than two or fewer micronutrients more effectively improves growth and other outcomes in micronutrient‐deficient children and adults. J Nutr 2009;139(5):1022‐30. doi:10.3945/jn.107.086199.

Ramakrishnan U, Nguyen P, Martorell R. Effects of micronutrients on growth of children under 5 y of age: meta‐analyses of single and multiple nutrient interventions. Am J Clin Nutr 2009; 89(1):191‐203. doi:10.3945/ajcn.2008.26862.

De‐Regil LM, Jeffards MD, Pena‐Rosas J. Powdered vitamins and minerals added to foods at the point‐of‐use reduces anaemia and iron deficiency in preschool‐ and school‐age children. Cochrane Database Syst Rev 2017, Issue 11. Art. No.: DC009666. DOI: 10.1002/14651858.CD009666.pub2.

Suchdev PS, Peña‐Rosas JP, De‐Regil LM. Multiple micronutrient powders for home (point‐of‐use) fortification of foods in pregnant women. Cochrane Database Syst Rev 2015, Issue 6. Art. No.: CD011158. doi:10.1002/14651858.CD011158.pub2.

De‐Regil LM, Suchdev PS, Vist GE, Walleser S, Peña‐Rosas JP. Home fortification of foods with multiple micronutrient powders for health and nutrition in children under two years of age. Cochrane Database Syst Rev 2011, Issue 9. Art. No.: CD008959.

De‐Regil LM, Jefferds MED, Peña‐Rosas JP. Point‐of‐use fortification of foods with micronutrient powders containing iron in children of preschool and school age. Cochrane Database Syst Rev 2017, Issue 11. Art. No.: CD009666. doi:10.1002/14651858.CD009666.pub2.

De‐Regil LM, Suchdev PS, Vist GE, Walleser S, Pena‐Rosas J. Use of a powder mix of vitamins and minerals to fortify complementary foods immediately before consumption and improve health and nutrition in children under two years of age. Cochrane Database Syst Rev 2011, Issue 9. Art. No.: CD008959. doi:10.1002/14651858.CD008959.

Salam RA, MacPhail C, Das JK, Bhutta ZA. Effectiveness of micronutrient powders (MNP) in women and children. BMC Public Health 2013;13(3):S22. doi:10.1186/1471‐2458‐13‐S3‐S22.

**Lipid‐based nutrient supplementation:**

Das JK, Salam RA, Weise PZ, et al. Provision of preventive lipid‐based nutrient supplements given with complementary foods to infants and young children 6 to 23 months of age for health, nutrition and developmental outcomes. 2017 Cochrane (review protocol).

**Large‐scale food fortification:**

Eichler K, Wieser S, Ruthemann I, Urs B. Effects of micronutrient‐fortified milk and cereal foods for infants and children: a systematic review. BMC Public Health 2012, 12:506. doi.org/10.1186/1471‐2458‐12‐506.

Ramakrishnan U, Goldenberg T, Allen LH. Do multiple micronutrient interventions improve child health, growth, and development? J Nutr. 2011, 141(11):2066‐75. doi:10.3945/jn.111.146845.

Das JK, Salam RA, Kumar R, Bhutta ZA. Micronutrient fortification of food and its impact on woman and child health: a systematic review. Syst Rev 2013, Issue 2. doi:10.1186/2046‐4053‐2‐67.

Keats EC, Neufeld L, Garrett, G, Bhutta, ZA. Large‐Scale Fortification: What Works in Low and Middle Income Settings? Evidence from a Systematic Review and Meta‐Analysis (in preparation).

Matsuyama M, Harb T, David M et al. Effect of fortified milk on growth and nutritional status in young children: a systematic review and meta‐analysis. Public Health Nutr. 2017, 20(7):1214‐1225. doi: 10.1017/S1368980016003189.

## 2 MEDLINE Search Strategy

Population:

1. exp infant/ or exp child/ or exp newborn/ or exp child, preschool/ or exp pediatrics/ or (infant* or neonat* or baby* or babies or child*or newborn* or “newborn infant*” or girl* or boy* or toddler* or preschool* or “pre school*” or pre‐school* or “preschool child*” or kindergarten* or kindergarden* or “nursery school” or “day care*” NOT adult* or paediatric* or pediatric* or under‐five* or “under five*” or “under 5” or under‐5 or “5 and under” or “five and under” or “under the age of five” or “under the age of 5”).tw,kf

Single or Multiple Micronutrient Supplementation:

2. Micronutrients/ or vitamins/ or minerals/ or exp iron/ or exp iron compounds/ or iron, dietary/ or vitamin A/ or exp iodine/ or exp zinc or exp zinc compounds/ or exp vitamin D/ or (micronutrient* or multinutrient* or multi‐nutrient* or “multi*nutrient” or “multimicro‐nutrient*” or “multimicronutrient*” or multivitamin* or “multi‐vitamin*” or multimineral* or “multi‐mineral*” or MMN or “multiple micro nutrient*” or “multiple micronutrient” or micro‐nutrient* or “essential vitamins*” or minerals* or “vitamin d” or “hydroxyvitamin d” or vitamin‐d or “25 hydroxyvitamin d” or “25 hydroxy‐vitamin d” or “25‐hydroxyvitamin d” or “25‐hydroxy‐vitamin d” or “25‐hydroxyvitamin d” or 25ohd or “25‐oh‐vitamin d” or 25‐ohd or “vitamin d2” or vitamin‐d2 or “25‐hydroxyvitamin d2” or “25‐hydroxy‐vitamin d2” or “vitamin d3” or vitamin‐d3 or “25 hydroxyvitamin d3” or “25 hydroxyvitamin d3” or “25‐hydroxy‐vitamin d3” or calcidiol or calcifediol or “vitamin a” or “vitamin a1” or retinol* or retinal* or Retinaldehyde or retinoid or Retinoids or retinoic or beta‐carotene or “beta carotene” or iron or ferr* compounds or “dietary iron” or zinc or “zn” or “zinc acetate” or “zn acetate” or “zn sulfate” or “zn oxide” or iodine or “iod* compounds” or “ferr* compounds” or “dietary iron”).tw,kf

3. Dietary supplements/ or tablets/ or ((supplement* or neutraceutical* or nutraceutical* or nutriceutical* or tablet* or syrup* or drop*).tw,kf)

4. 1 AND 2 AND 3

Lipid‐Nutrient Supplementation:

5. exp Lipids/ or omega‐3/ or (lipid* or soy* or peanut* or whey* or sesame* or cashew* or chickpea* or oils or protein* or butter* or fat* or “alpha‐linolenic acid” or “docosahexaenoic acids” or “eicosapentaeonic acid”).tw,kf

6. Dietary supplements/ or ((“lipid based” or “lipid‐based nutri*” or enrich* or emuls* or powder* or spread* or paste* or LNS*1 or iLiNS or supplement* or neutraceutical* or nutraceutical* or nutriceutical* or Nutributter* or Plumpy* or PlumpyNut or “ready to use” or “ready‐to‐use therapeutic food” or RUSF or RUTF).tw,kf)

7. 1 AND 5 AND 6

Large‐Scale Fortification:

8. exp Food/ or “Food Supply”/ or exp “Agricultural Crops”/ or exp Salts/ or exp “Fish Products”/ or exp “Soy Foods” or exp Cereals/ or exp “Dietary Carbohydrates”/ or exp Oryza/ or exp Milk/ or exp Bread/ or exp Beverages/ or exp Yogurt/ or exp Margarine/ or exp Cheese/ or exp “Zea mays”/ or exp Condiments/ or exp Triticum/ or exp Spices/ or exp “Dietary Fats”/ or exp “Dairy Products”/ or (Food* or “staple foods” or crop* or flour* or salt* or sauce*or cereal* or sugar* or rice* or milk* or bread* or oil* or beverage* or yogurt* or margarine* or cheese* or maize*or corn* or wheat* or “durum wheat*” or “sorghum vulgare” or sorghum or condiment* or spices* or “curry powder*” or fat* or dairy*).tw,kf

9. Micronutrients/ or vitamins/ or minerals/ or exp iron/ or exp iron compounds/ or iron, dietary/or vitamin A/ or exp iodine/ or exp zinc/ or exp zinc compounds/ or exp vitamin D/ or exp folic acid/ or (micronutrient* or multinutrient* or multi‐nutrient* or “multi*nutrient” or “multimicro‐nutrient*” or “multimicronutrient*” or multivitamin* or “multi‐vitamin*” or multimineral* or “multi‐mineral*” or MMN or “multiple micro nutrient*” or “multiple micronutrient” or micro‐nutrient* or “essential vitamins*” or minerals* or “vitamin d” or “hydroxyvitamin d” or vitamin‐d or “25 hydroxyvitamin d” or “25 hydroxy‐vitamin d” or “25‐hydroxyvitamin d” or “25‐hydroxy‐vitamin d” or “25‐hydroxyvitamin d” or 25ohd or “25‐oh‐vitamin d” or 25‐ohd or “vitamin d2” or vitamin‐d2 or “25‐hydroxyvitamin d2” or “25 hydroxy‐vitamin d2” or “vitamin d3” or vitamind3 or “25 hydroxyvitamin d3” or “25 hydroxyvitamin d3” or “25‐hydroxy‐vitamin d3” or calcidiol or calcifediol or “vitamin a” or “vitamin a1” or retinol* or retinal* or Retinaldehyde or retinoid or Retinoids or retinoic or beta‐carotene or “beta carotene” or iron or ferr* compounds or “dietary iron” or zinc or “zn” or “zinc acetate” or “zn acetate” or “zn sulfate” or “zn oxide” or iodine or “iod* compounds” or “ferr* compounds” or “dietary iron” or “folic acid” or folate*).tw,kf.

10. (“supplemented food*” or “large scale fortif*” or enrich* or fortif*).tw,kf.

11. 1 AND 8 AND 9 AND 10

Targeted Fortification for Infants and Young Children:

12. exp “infant food”/ or milk substitutes/ or infant formula/ or soy milk/ or (“baby formula” or “artificial milk” or “complementary food*” or “blended food*” or “infant cereal” or “baby food*” or milk* or cereal* or porridge* or paste*).tw,kf

13. (“targeted fortification” or enrich* or fortif* or “supplemented food*”).tw,kf

14. 1 AND 12 AND 13

Point‐Of‐Use Fortification with Micronutrient Powders:

15. Powders/ or vitamins/ or minerals/ or micronutrients/ or (“sprinkles powder” or Sprinkle* or powder* or foodlet* or “foodlet‐based” or “crushable nutritabs” or “micronutrient powder” or “multiple micronutrient powder” or mnp).tw,kf

16. (“Point‐of‐use” or “home fortification” or “food fortif*” or enrich* or fortif*).tw,kf

17. 1 AND 15 AND 16

18. Developing Countries/ or (“developing country” or “developing countries” or “developing nation” or “developing nations” or “developing population” or “developing populations” or “developing world” or “less developed country” or “less developed countries” or “less developed nation” or “less developed nations” or “less developed population” or “less developed populations” or “less developed world” or “lesser developed country” or “lesser developed countries” or “lesser developed nation” or “lesser developed nations” or “lesser developed population” or “lesser developed populations” or “lesser developed world” or “under developed country” or “under developed countries” or “under developed nation” or “under developed nations” or “under developed population” or “under developed populations” or “under developed world” or “underdeveloped country” or “underdeveloped countries” or “underdeveloped nation” or “underdeveloped nations” or “underdeveloped population” or “underdeveloped populations” or “underdeveloped world” or “middle income country” or “middle income countries” or “middle income nation” or “middle income nations” or “middle income population” or “middle income populations” or “low income country” or “low income countries” or “low income nation” or “low income nations” or “low income population” or “low income populations” or “lower income country” or “lower income countries” or “lower income nation” or “lower income nations” or “lower income population” or “lower income populations” or “underserved country” or “underserved countries” or “underserved nation” or “underserved nations” or “underserved population” or “underserved populations” or “underserved world” or “under served country” or “under served countries” or “under served nation” or “under served nations” or “under served population” or “under served populations” or “under served world” or “deprived country” or “deprived countries” or “deprived nation” or “deprived nations” or “deprived population” or “deprived populations” or “deprived world” or “poor country” or “poor countries” or “poor nation” or “poor nations” or “poor population” or “poor populations” or “poor world” or “poorer country” or “poorer countries” or “poorer nation” or “poorer nations” or “poorer population” or “poorer populations” or “poorer world” or “developing economy” or “developing economies” or “less developed economy” or “less developed economies” or “lesser developed economy” or “lesser developed economies” or “under developed economy” or “under developed economies” or “underdeveloped economy” or “underdeveloped economies” or “middle income economy” or “middle income economies” or “low income economy” or “low income economies” or “lower income economy” or “lower income economies” or “low gdp” or “low gnp” or “low gross domestic” or “low gross national” or “lower gdp” or “lower gnp” or “lower gross domestic” or “lower gross national” or lmic or lmics or “third world” or “lami country” or “lami countries” or “transitional country” or “transitional countries” or Africa or Asia or “Caribbean Region” or “West Indies” or “South America” or “Latin America” or “Central America” or Afghanistan or Albania or Algeria or Angola or Argentina or Armenia or Armenian or Azerbaijan or Bangladesh or Benin or Byelarus or Byelorussian or Belarus or Belorussian or Belorussia or Belize or Bhutan or Bolivia or Bosnia or Herzegovina or Hercegovina or Botswana or Brazil or Bulgaria or “Burkina Faso” or “Burkina Fasso” or Burundi or Urundi or Cambodia or “Khmer Republic” or Kampuchea or Cameroon or Cameroons or Cameron or Camerons or “Cape Verde” or “Central African Republic” or Chad or China or Colombia or Comoros or “Comoro Islands” or Comores or Mayotte or Congo or Zaire or “Costa Rica” or “Cote d'Ivoire” or “Ivory Coast” or Cuba or Djibouti or “French Somaliland” or Dominica or “Dominican Republic” or “East Timor” or “East Timur” or “Timor Leste” or Ecuador or Egypt or “El Salvador” or Eritrea or Ethiopia or Fiji or Gabon or “Gabonese Republic” or Gambia or Gaza or “Georgia Republic” or “Georgian Republic” or Ghana or “Gold Coast” or Grenada or Guatemala or Guinea or Guiana or Guyana or Haiti or Honduras or India or Maldives or Indonesia or Iran or Iraq or “Isle of Man” or Jamaica or Jordan or Kazakhstan or Kazakh or Kenya or Kiribati or Korea or Kosovo or Kyrgyzstan or Kirghizia or “Kyrgyz Republic” or Kirghiz or Kirgizstan or “Lao PDR” or Laos or Lebanon or Lesotho or Basutoland or Liberia or Libya or Macedonia or Madagascar or “Malagasy Republic” or Malaysia or Malaya or Malay or Sabah or Sarawak or Malawi or Nyasaland or Mali or “Marshall Islands” or Mauritania or Mauritius or Mexico or Micronesia or “Middle East” or Moldova or Moldovia or Moldovian or Mongolia or Montenegro or Morocco or Mozambique or Myanmar or Myanma or Burma or Namibia or Nepal or Nicaragua or Niger or Nigeria or Pakistan or Palau or Palestine or Panama or Paraguay or Peru or Philippines or Philipines or Phillipines or Phillippines or Romania or Rumania or Roumania or Russia or Russian or Rwanda or Ruanda or “Saint Lucia” or “St Lucia” or “Saint Vincent” or “St Vincent” or Grenadines or Samoa or “Samoan Islands” or “Navigator Island” or “Navigator Islands” or “Sao Tome” or Senegal or Serbia or Montenegro or Seychelles or “Sierra Leone” or “Sri Lanka” or Ceylon or “Solomon Islands” or Somalia or Sudan or Suriname or Surinam or Swaziland or Syria or Tajikistan or Tadzhikistan or Tadjikistan or Tadzhik or Tanzania or Thailand or Togo or “Togolese Republic” or Tonga or Tunisia or Turkey or Turkmenistan or Turkmen or Uganda or Ukraine or USSR or “Soviet Union” or “Union of Soviet Socialist Republics” or Uzbekistan or Uzbek or Vanuatu or Venezuela or Vietnam or “Viet Nam” or “West Bank” or Yemen or Yugoslavia or Zambia or Zimbabwe or Rhodesia).tw,kf

19. 4 OR 7 OR 11 OR 14 OR 17

20. 19 AND 18

21. Limit 20 to yr=“1995‐2017”
